# Chylous Ascites After Robotic Retroperitoneal Lymph Node Dissection Resolved After 80 Days of Conservative Management: A Case Report

**DOI:** 10.7759/cureus.107852

**Published:** 2026-04-27

**Authors:** Markus Angerer, Niclas Flechtenmacher, Christian Wülfing, Klaus-Peter Dieckmann

**Affiliations:** 1 Department of Urology, Asklepios Klinik Altona, Hamburg, DEU

**Keywords:** chylous ascites, parenteral nutrition, retroperitoneal lymph node dissection, robotic surgery, seminoma, testicular cancer

## Abstract

Chylous ascites (CA) is the accumulation of chylous lymphatic fluid within the abdominal cavity, usually resulting from laceration of the cisterna chyli or one of its major tributaries during retroperitoneal surgery. If left untreated, CA may become life-threatening. Current treatment recommendations favor a stepwise approach, beginning with conservative measures, followed by percutaneous interventions and, in refractory cases, surgical shunting procedures.

Conservative management typically consists of dietary restriction of long-chain triglycerides, escalation to total parenteral nutrition (TPN), and adjunctive pharmacotherapy with somatostatin analogues. In most reported cases, these measures result in cessation of chylous leakage within several days.

We report a case of CA after robotic-assisted retroperitoneal lymph node dissection (RPLND) in a 35-year-old male with testicular seminoma. Conservative management included paracentesis with drain insertion, TPN, and pharmacotherapy with octreotide. Ascitic fluid analysis confirmed CA with a triglyceride level of 420 mg/dL. Because chylous leakage did not cease after 60 days of hospital-based management, home-based TPN via a central venous port was initiated. Chylous leakage finally resolved after 80 days of conservative treatment. One year later, the patient remained well and had experienced no recurrence of either CA or seminoma.

This case demonstrates that conservative treatment of CA may occasionally require a prolonged course and considerable patience from both the patient and the treating team.

## Introduction

Chylous ascites (CA) is a rare but potentially life-threatening complication of retroperitoneal surgery [1]. It may result either from intraoperative laceration of the cisterna chyli (a dilated sac at the lower end of the thoracic duct, usually located dorsal to the right renal artery), which is usually located dorsal to the right renal artery, or from disruption of one of its major tributaries arising from the portal or splanchnic lymphatic system (the network of lymphatic vessels draining the abdominal organs) [2]. Clinically, CA typically presents with abdominal discomfort, progressive abdominal distension due to fluid accumulation, and, in some cases, dyspnea [3]. The diagnosis is established by paracentesis (needle aspiration of abdominal fluid), demonstrating a whitish-yellow, milky, odorless, lipid-rich, and alkaline fluid with elevated triglyceride levels (typically >110 mg/dL).

Current treatment recommendations advocate a stepwise approach that begins with conservative measures, including dietary modification restricting long-chain triglycerides (which are transported through lymphatic channels, unlike medium-chain triglycerides that enter the portal venous system directly) and pharmacotherapy, proceeds to percutaneous interventions when necessary, and ultimately includes surgical procedures in refractory cases [4,5]. Approximately 70% of cases are successfully managed with conservative treatment alone, with an average time to resolution of about 11 days [6]. The present case illustrates that, despite this generally favorable experience, conservative treatment may occasionally be prolonged and frustrating for both the patient and the surgeon.

## Case presentation

Initial diagnosis and primary treatment

A 35-year-old male with no significant past medical or surgical history underwent left radical orchiectomy for a 4.8 cm seminoma with angiolymphatic invasion (pT2L1V1). Staging investigations showed no evidence of metastatic disease, and clinical stage IB was assumed. The patient was therefore managed with surveillance.

Recurrence and surgical intervention

**Figure 1:**
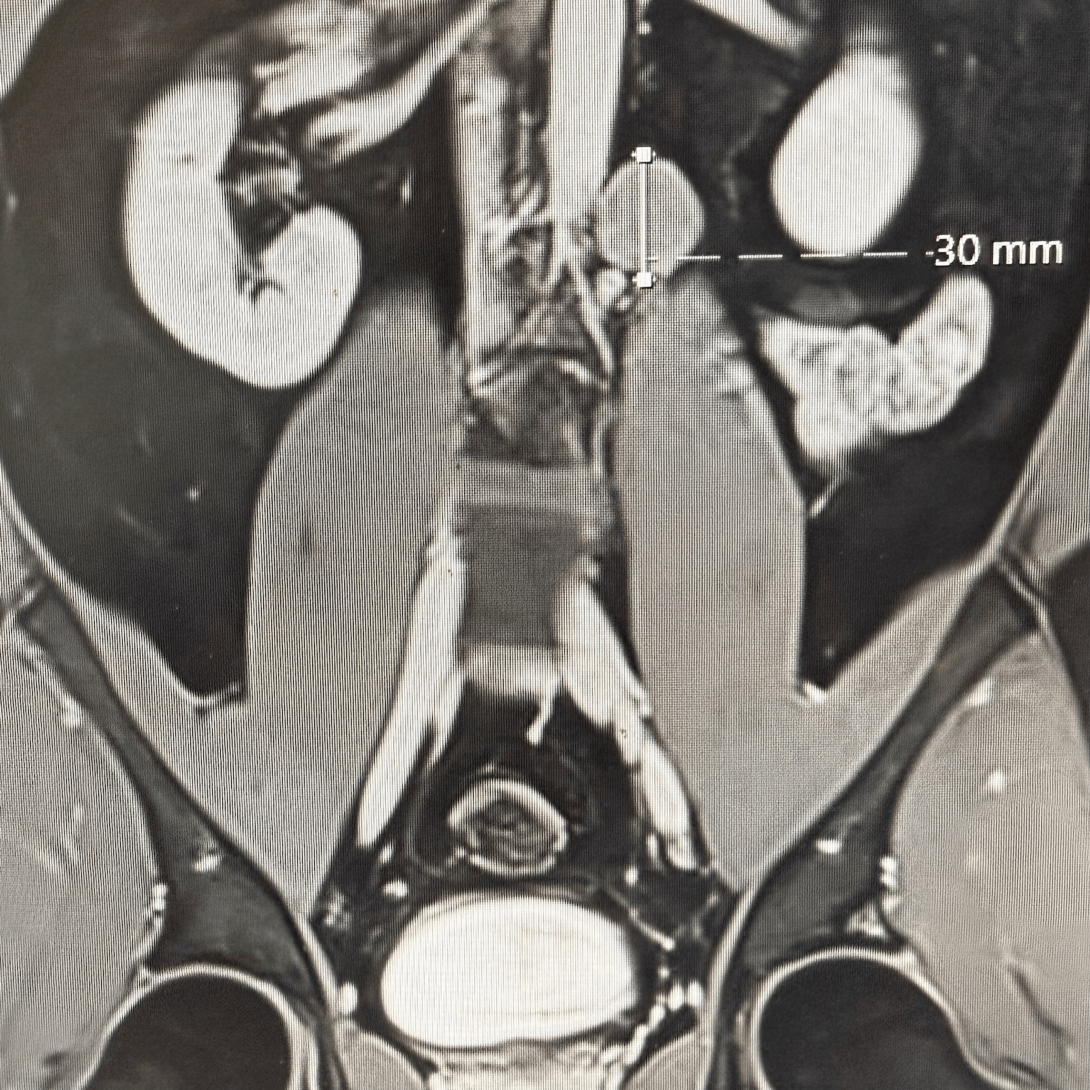
Preoperative MRI showing the location of the 3 cm para-aortic lymphadenopathy.

Four years after the initial diagnosis, follow-up magnetic resonance imaging demonstrated a 3 cm para-aortic lymphadenopathy suspicious for metastatic recurrence (Figure 1).

One month later, the patient underwent robotic-assisted unilateral retroperitoneal lymph node dissection (RPLND). Histopathological examination confirmed a 4.5 cm seminoma metastasis in one of 10 harvested para-aortic lymph nodes. No surgical drain was placed at the completion of the procedure. The immediate postoperative course was uneventful, and the patient was discharged on postoperative day (POD) four with a plan to begin adjuvant carboplatin chemotherapy within three weeks.

Postoperative complication and diagnosis

**Figure 2:**
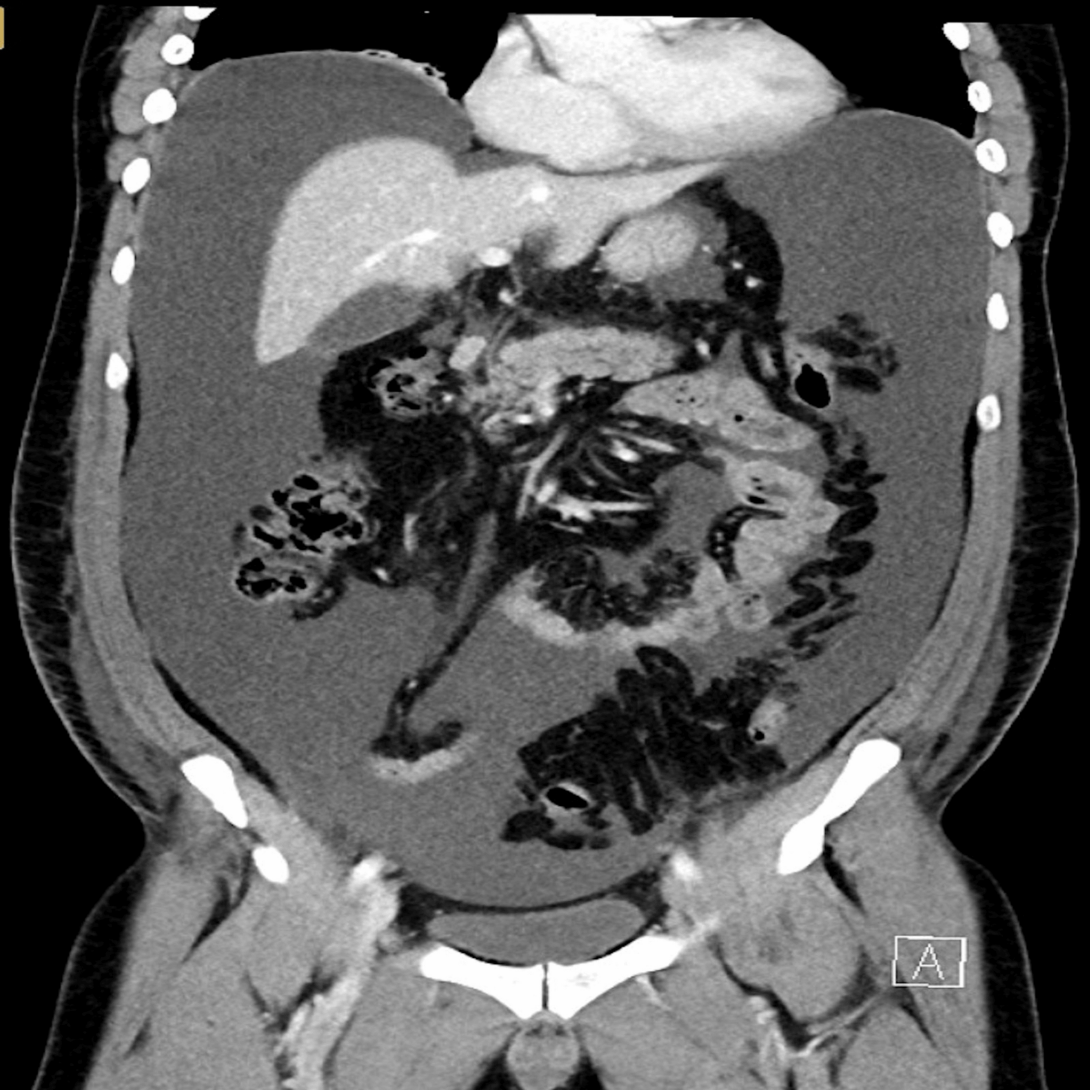
Abdominal computed tomography on postoperative day 23 showed marked four-quadrant ascites.

On POD 23, the patient was readmitted with progressive abdominal distension and mild diffuse abdominal discomfort. Physical examination revealed a distended, non-tender abdomen with shifting dullness. The patient was hemodynamically stable with no fever or signs of peritonitis. Computed tomography demonstrated marked four-quadrant ascites (Figure 2).

Ultrasound-guided paracentesis yielded 5.7 L of milky whitish-yellow fluid, confirming chylous ascites. Fluid analysis revealed a triglyceride level of 420 mg/dL, total protein of 3.8 g/dL, white blood cell count of 180 cells/μL with predominant lymphocytes, and negative bacterial culture. A percutaneous drain was placed in the right lower quadrant for ongoing decompression.

Conservative management

Initial treatment consisted of inpatient conservative management with total parenteral nutrition (TPN) administered through peripheral venous access. Initial drain output on POD 24 was 2,500 mL, decreasing to 650 mL on POD 25, then showing significant daily variability ranging from 350 to 1,250 mL during the first week. Chylous output showed fluctuating patterns with volumes ranging from 200 to 1,200 mL/day during weeks two to four (POD 31-52). Two attempts to reintroduce oral intake on POD 30 (730 mL output) and POD 38 (200 mL output) resulted in variable drainage patterns without a clear immediate correlation to oral intake attempts and immediate recurrence of milky drainage. A 10-day course of subcutaneous octreotide (100 μg twice daily) was then administered from POD 40 to POD 50. During this period, output ranged from 300 to 700 mL/day, with some improvement noted by POD 55 (150 mL). Although the output transiently decreased, a third oral challenge again resulted in recurrent chylous excretion.

Home-based TPN and resolution

As the leak remained refractory despite prolonged inpatient treatment, and after multidisciplinary team discussion involving surgery, oncology, nutrition, and interventional radiology, the decision was made to continue conservative management with home-based TPN rather than proceed to invasive intervention. This approach was chosen to allow continued observation for spontaneous resolution while avoiding the risks and potential complications of surgical intervention or lymphatic sclerotherapy, particularly given that the patient required adjuvant chemotherapy and any additional invasive procedure could further delay cancer treatment. The patient was counseled extensively about this prolonged conservative approach and agreed to proceed.

**Figure 3:**
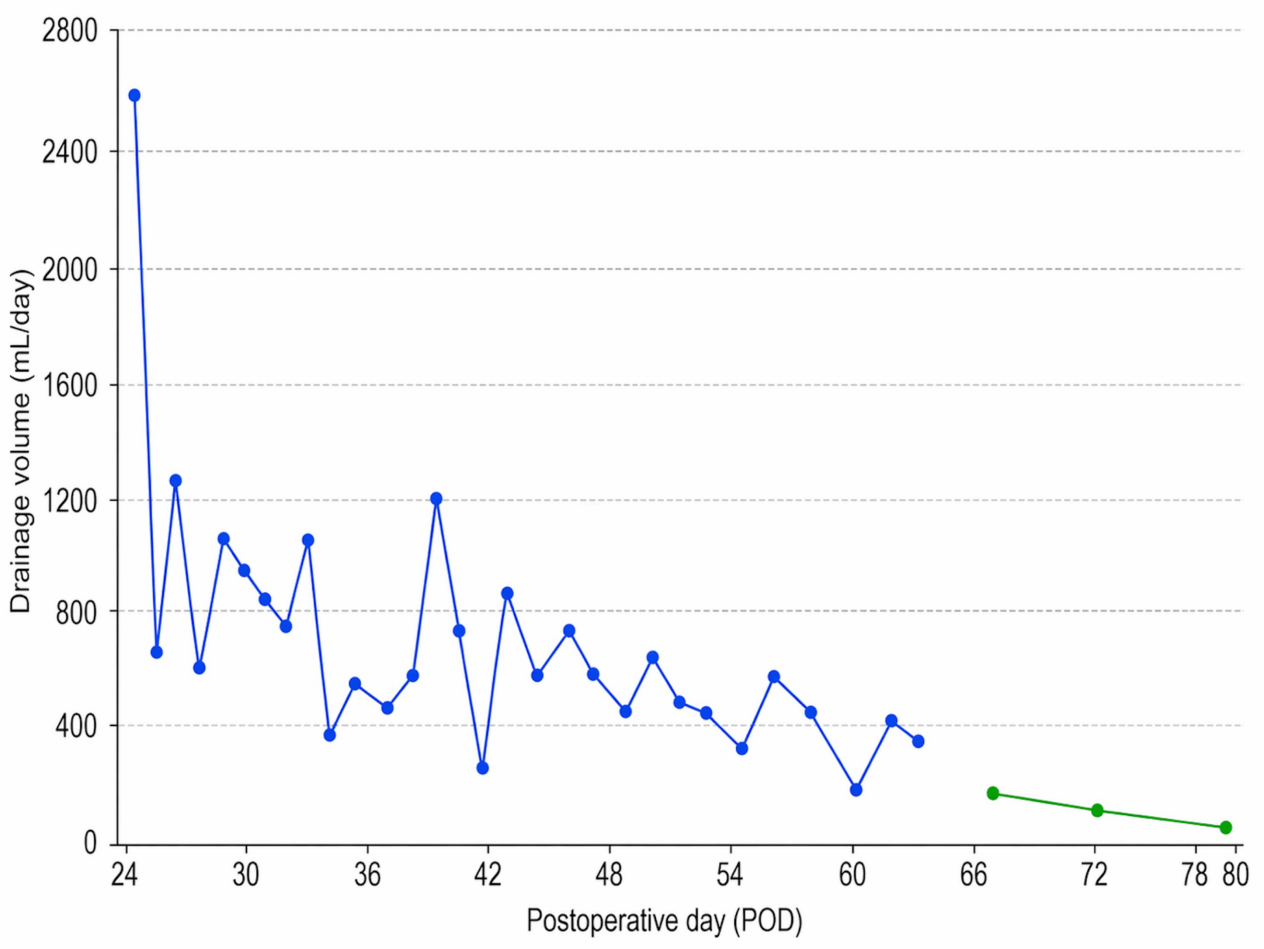
Postoperative drainage output over time during conservative management of chylous ascites. Daily drainage volume (mL/day) is shown according to postoperative day (POD). The blue line represents the period of in-hospital conservative management, consisting of total parenteral nutrition (TPN) and a 10-day course of subcutaneous octreotide. The green line represents the subsequent period of long-term home-based TPN. Drain placement was performed on POD 24.

Thus, long-term home-based TPN was initiated. A central venous port was implanted on POD 57, and from POD 61 onward, the patient received overnight TPN infusions at home while remaining nil per os except for water and unsweetened tea. Daily drain output gradually decreased, and progressive improvement was observed with output decreasing to 150 mL on POD 66 and 85 mL on POD 72. During the period of prolonged TPN, the patient remained nutritionally stable, with a body weight of 76 kg and serum albumin levels maintained at 3.4-3.6 g/dL throughout the TPN period, indicating that prolonged conservative management was well tolerated. On POD 80, drainage decreased substantially to less than 30 mL/day, and complete cessation occurred on POD 81. Figure 3 illustrates the daily drainage volumes plotted against postoperative days, demonstrating the fluctuating but generally improving trajectory of chylous output over the 80-day treatment period.

Oral intake was cautiously resumed on POD 83 without recurrence, and the abdominal drain was removed on POD 86.

Follow-up

The planned adjuvant chemotherapy was delayed and ultimately administered as two cycles of carboplatin (area under the curve (AUC): 7) on POD 105 and POD 134 without significant toxicity. The central venous port was removed five months after RPLND. At one-year follow-up, the patient remained in complete remission without recurrence of CA and without residual sequelae related to either the surgical complication or its treatment.

## Discussion

CA remains incompletely understood, largely because of its rarity and the broad range of therapeutic options that have been reported. It most commonly occurs after retroperitoneal surgery, particularly major visceral or vascular procedures, although CA has also been described after RPLND in patients with urological and gynecological malignancies [2]. The first report of CA after RPLND for testicular cancer was published in 1971 [7]. The incidence after standard open RPLND has been reported to range from approximately 1% to 7% [8,9]. Interestingly, the frequency appears to be higher after robotic-assisted RPLND, with reported rates of 11% to 31% [10,11]. In our own series, five of 40 patients undergoing robotic RPLND developed CA (12.5%), including the patient presented here [12]. The reason for the apparently higher rate after robotic surgery remains unclear, although less rigorous clipping or ligation of lymphatic vessels compared with open surgery has been proposed as a possible explanation.

In a systematic review of CA treatment modalities, conservative management achieved a success rate of 69%, with a median time to resolution of 11 days [6]. The first step of conservative treatment is usually dietary restriction of long-chain triglycerides. The rationale is that long-chain triglycerides are transported through the intestinal lymphatic channels to the cisterna chyli, whereas medium-chain triglycerides are absorbed as free fatty acids and enter the portal venous system without passing through the lymphatic circulation. If this measure fails, the next step usually consists of a nil per os strategy combined with TPN. Pharmacological support may include diuretics such as furosemide and somatostatin analogues such as octreotide. Octreotide is thought to reduce chyle leakage indirectly by lowering portal pressure. Retrospective studies have suggested that this pharmacological approach may shorten the duration of chyle leakage by up to 50% [13].

If conservative treatment fails, escalation options include percutaneous procedures such as lymphangiographic sclerotherapy and, in refractory cases, surgical management with direct oversewing of the chyle leak or placement of artificial shunt systems such as the Denver shunt to divert ascitic fluid into the central venous system [14-16]. However, both percutaneous and surgical interventions are associated with substantial failure rates, and secondary surgery in particular may itself be complicated. Accordingly, conservative treatment remains the preferred initial strategy, and more invasive options should generally be reserved for cases in which conservative measures have clearly failed [6]. In the present case, the decision to continue conservative management for an extended period was made collaboratively within a multidisciplinary team. Key considerations included the following: (1) the gradual but consistent downward trend in drain output over time, suggesting ongoing healing; (2) the patient's need for adjuvant chemotherapy, which could be complicated by additional surgical intervention; (3) the documented risks and failure rates of both percutaneous lymphatic sclerotherapy and surgical shunting procedures; (4) the patient's hemodynamic stability and tolerance of home-based TPN; and (5) the patient's informed preference to avoid additional invasive procedures if possible. While the prolonged duration of conservative therapy (80 days) significantly delayed chemotherapy initiation, the oncology team deemed this acceptable given the patient's good performance status and the relatively favorable prognosis of his disease.

Because CA is rare, the maximum duration for which conservative treatment should be continued before invasive intervention is not well defined. In the recent review cited above, the median time to resolution with conservative therapy was 11 days [6]. Most reported patients recovered within a few days, as was also the case for four other patients from our own series. Nevertheless, more prolonged courses have been described. One patient from Morocco required 30 days to recover [17]. Sporadic cases with durations of six to eight weeks, and even as long as 39 weeks, have also been reported [5,8,18]. The present case, therefore, represents a particularly prolonged course of conservative treatment, although not the longest reported. In our patient, TPN ultimately led to resolution after 80 days.

Of note, octreotide did not appear to provide a durable benefit in the present case. Although conclusions cannot be drawn from a single patient, this observation is consistent with a report from Indiana University, where this treatment was abandoned because of its apparent lack of efficacy in patients with testicular cancer [18]. Low-dose radiotherapy was first reported as a possible treatment for CA in the 1970s [19], but it has not achieved broad acceptance, even though a total dose of 8 Gy has been used successfully in CA after gastrointestinal surgery. More recently, the feasibility of radiotherapy for CA after RPLND in testicular cancer has again been documented [20]. Therefore, despite understandable concerns regarding irradiation in young germ cell tumor patients, this modality may be worth considering in otherwise refractory cases before proceeding to invasive surgery. However, radiotherapy was not considered in our patient due to the ongoing downward trend in chylous output and the expectation that conservative measures would eventually succeed.

## Conclusions

CA is a rare but potentially serious complication of retroperitoneal surgery in patients with testicular cancer. Conservative treatment usually proceeds in a stepwise fashion, beginning with dietary fat restriction and progressing to nil per os management with TPN, with or without somatostatin analogue therapy. The present case demonstrates that conservative management may occasionally require a very prolonged course and substantial patience from both the patient and the treating team. The decision to continue conservative therapy beyond typical timeframes should be made within a multidisciplinary setting, weighing factors such as clinical stability, trend in output volumes, timing of required oncological treatment, and risks of alternative interventions. In refractory cases, home-based TPN, supported by ambulatory care services, may provide a feasible means of extending conservative management beyond the inpatient setting while maintaining quality of life and avoiding the morbidity associated with invasive procedures.
